# Daboxin P, a Major Phospholipase A2 Enzyme from the Indian *Daboia russelii russelii* Venom Targets Factor X and Factor Xa for Its Anticoagulant Activity

**DOI:** 10.1371/journal.pone.0153770

**Published:** 2016-04-18

**Authors:** Maitreyee Sharma, Janaki Krishnamurthy Iyer, Norrapat Shih, Munmi Majumder, Venkata Satish Kumar Mattaparthi, Rupak Mukhopadhyay, Robin Doley

**Affiliations:** 1 Department of Molecular Biology and Biotechnology, Tezpur University, Tezpur-784028, Assam, India; 2 Department of Biological Sciences, Faculty of Science, National University of Singapore, Singapore, Singapore; Russian Academy of Sciences, Institute for Biological Instrumentation, RUSSIAN FEDERATION

## Abstract

In the present study a major protein has been purified from the venom of Indian *Daboia russelii russelii* using gel filtration, ion exchange and Rp-HPLC techniques. The purified protein, named daboxin P accounts for ~24% of the total protein of the crude venom and has a molecular mass of 13.597 kDa. It exhibits strong anticoagulant and phospholipase A_2_ activity but is devoid of any cytotoxic effect on the tested normal or cancerous cell lines. Its primary structure was deduced by N-terminal sequencing and chemical cleavage using Edman degradation and tandem mass spectrometry. It is composed of 121 amino acids with 14 cysteine residues and catalytically active His48 -Asp49 pair. The secondary structure of daboxin P constitutes 42.73% of α-helix and 12.36% of β-sheet. It is found to be stable at acidic (pH 3.0) and neutral pH (pH 7.0) and has a Tm value of 71.59 ± 0.46°C. Daboxin P exhibits anticoagulant effect under *in-vitro* and *in-vivo* conditions. It does not inhibit the catalytic activity of the serine proteases but inhibits the activation of factor X to factor Xa by the tenase complexes both in the presence and absence of phospholipids. It also inhibits the tenase complexes when active site residue (His48) was alkylated suggesting its non-enzymatic mode of anticoagulant activity. Moreover, it also inhibits prothrombinase complex when pre-incubated with factor Xa prior to factor Va addition. Fluorescence emission spectroscopy and affinity chromatography suggest the probable interaction of daboxin P with factor X and factor Xa. Molecular docking analysis reveals the interaction of the Ca^+2^ binding loop; helix C; anticoagulant region and C-terminal region of daboxin P with the heavy chain of factor Xa. This is the first report of a phospholipase A_2_ enzyme from Indian viper venom which targets both factor X and factor Xa for its anticoagulant activity.

## Introduction

Haemostasis, one of the most important physiological processes of vertebrates, involves four crucial steps for sustaining equilibrium, namely, (i) vasoconstriction, to reduce blood flow from the site of injury (ii) platelet activation, aggregation and adherence leading to the platelet plug formation at the injured site (iii) initiation of coagulation cascade involving the extrinsic, intrinsic and the common pathway, forming fibrin mesh on the platelet plug and (iv) fibrinolysis leading to the dissolution of the clot formed, in order to restore normal blood flow [1]. Any malfunction in this vital process leads to two major pathophysiological conditions, haemorrhage or thrombosis, at large. Interestingly, the components of the haemostatic system of the prey/victim are one of the most vulnerable targets in snake envenomation. Owing to this, there has been a quest amongst the venom researchers to unfold the underlying mechanism and explore the therapeutic potentiality of these venom proteins since the last few decades. Recent trend of research reveals a pursuit for direct inhibitors of factor Xa (FXa) and thrombin from the venom of snakes and saliva of hematophagous animals as one of the most thrived components for anticoagulant and antithrombotic drug discovery [2–7].

*Daboia russelii russelii (Daboia r*. *russelii)*, a venomous snake of the Indian subcontinent, contains an intricate blend of various biologically active protein families. Consumptive coagulopathy is one of the most manifested systemic pathological condition observed in viper envenomated victims [8]. The procoagulant venom proteins like RVV-X and RVV-V activators accelerate the blood coagulation cascade and the simultaneous down regulation of activated protein C causes severe depletion of the coagulation factors thereby depriving the victim of the serine proteases shortly [8]. This scenario is further worsened by the anticoagulant and haemorrhagic components like phospholipase A_2_ (PLA_2_) enzymes, L-amino acid oxidase (LAAO) and metalloproteases leading to hypovolemic shock and even death [9].

PLA_2_ enzymes are one of the major protein families in Russell’s viper proteome with various isoforms [10,11]. Although the members of this family share a highly conserved structural and catalytic scaffold, plethora of pharmacological activities are reported among the isoforms apart from prey digestion [12–15]. The subtle differences in their activities is mainly attributed to the presence of various pharmacological sites (responsible for non-enzymatic activity via protein-protein interaction) which are distinct from the catalytic site (responsible for enzymatic activity via protein-phospholipid interaction) [16]. The presence of minor amino acid variations in the exposed regions of these isoforms one of the vital causes of diverse pharmacological sites [3]. The ability of the snake venom PLA_2_ enzymes to inhibit blood coagulation was first reported by Boffa & Boffa in *Vipera berus* venom [17]. Henceforth, many anticoagulant PLA_2_ enzymes have been reported from snake venom by several researchers. These enzymes are classified into strong, weak and non-anticoagulant based on the concentration required to delay clot formation and amino acid residues in the predicted anticoagulant region (54^th^ to 77^th^ residues) [16,18]. These enzymes act either by hydrolyzing the procoagulant phospholipids or target the coagulation factors for its activity. CM-I and CM-II from *Naja nigricollis* and Vipoxin from *Vipera ammodtes ammodytes* hydrolyze the phospholipids required for the formation of the extrinsic and intrinsic tenase complex [19–21]. CM-IV from *Naja nigricollis*, AtxA from *Vipera ammodytes ammodytes*, CBc from *Crotalus durissus terrificus* and MtxII from *Bothrops asper*, target FXa of the prothrombinase complex and impede the conversion of prothrombin to thrombin [6,22]. While Nn-PLA_2_ from *Naja naja* and Nk-PLA_2_- β from *Naja kaouthia* target thrombin directly to exhibit anticoagulant effect [23,24]. On the other hand, VRV-PL-IIIb from *Daboia russelii*, CHA-E6b & CHA-E6a from *Crotalus horridus* and Tj-PLA_2_ from *Trimeresurus jerdoni* inhibit ADP and collagen induced platelet aggregation [25–27].

In the present study we have isolated and characterized a strong anticoagulant PLA_2_ enzyme, from the venom of Indian *Daboia russelii russelii*. Enzymatically it hydrolyses the phospholipids required for the formation of the tenase complexes and non-enzymatically it targets factor X (FX) and activated factor X (FXa) for exhibiting its anticoagulant activity. Hence, this purified anticoagulant PLA_2_ enzyme was named as daboxin P (*Dabo**ia russelii russelii* FX inhibitor PLA_2_ enzyme).

## Materials and Methods

### Crude venom and chemicals/reagents

Crude venom of *Daboia r*. *russelii* (1 gm) was purchased from Irula Snake Catchers Society, Tamil Nadu, India. Few individuals (~3–4 individuals) of each snake species are caught from the forest and captivated for nearly 3–4 weeks before venom extraction. During this period venom is milked from the snakes for 4–5 times before releasing back to the forest. None of the captivated snakes are harmed or killed. Secretory phospholipase A_2_ (sPLA_2_) assay kit was obtained from Cayman Chemical Company (MI, USA). 4-vinyl pyridine, hydroxylamine hydrochloride, Cyanogen bromide activated Sepharose® 4B and bovine plasma fibrinogen were purchased from Sigma-Aldrich (MO, USA). BNPS-skatole [2-(2-Nitrophenylsulfenyl)-3-methyl-3-bromoindolenine)] was purchased from Bioworld (OH, USA). The Edman sequencing reagents were purchased from Applied Biosystems chemicals (Foster city, CA). The rest of the reagents and chemicals were of analytical grade and obtained from Merck Millipore (MA, USA) or Sigma (MO, USA).

### Enzymes and chromogenic substrates

Serine proteases factor XIa (FXIa), factor IXa (FIXa), factor VIIa (FVIIa), factor X (FX), factor Xa (FXa) and Russell’s viper venom-X activator (RVV-X) were obtained from Haematologic Technologies Inc. (Vermont, USA), factor XIIa (FXIIa) was procured from Merck Calbiochem (Darmstadt, Germany), factor VIII (FVIII) from Creative Biomart (NY, USA), phospholipid blend from Avanti Polar Lipids Inc. (Alabama, USA) and tissue factor Innovin from Siemens (Murburg, Germany). The chromogenic substrates namely spectrozyme (MeSO_2_-D-CHG-Gly-Arg-pNA.AcOH) was purchased from Sekisui Diagnostics (MA, USA), rest of the substrates like S-2366 (pyroGlu-Pro-Arg-pNA•HCl), S-2302 (H-D-Pro-Phe-Arg-pNA•2HCl), S-2222 (Bz-IIe-Glu(γ-OR)-Gly-Arg-pNA•HCl), S-2765 (Z-D-Arg-Gly-Arg-pNA•2HCl)S-2238 (H-D-Phe-Pip-Arg-pNA•2HCl) and S-2288 (H-D-Ile-Pro-Arg-pNA•2HCl) were procured from Chromogenix (NJ,USA).

### Cell culture

Human embryonic kidney (HEK)-293 and Michigan Cancer Foundation (MCF) -7 cell lines were purchased from National Centre for Cell Science (Pune, India). Dulbecco modified Eagle’s media (DMEM) was obtained from Himedia (Mumbai, India), 3-(4,5-dimethylthiazol-2-yl)-2,5-diphenyltetrazolium bromide (MTT) and trypan blue from Sigma Aldrich (Mo, USA), fetal bovine serum (FBS), streptomycin-penicillin (Strep-Pen), trypsin/EDTA solution and dimethyl sulfoxide (DMSO) were procured from Thermo Fischer (MA, USA).

### Columns

Hiload^TM^ 16/600 Superdex 75 prep grade and Hiprep CM FF 16/10 columns were purchased from GE Healthcare life Sciences (Bucks, UK). Jupiter C_18_ column was procured from Phenomenex (CA, USA) and Hypersil Gold C_18_ column was obtained from Thermo Scientific (MA, USA).

### Purification of the major anticoagulant protein

Crude *Daboia r*. *russelii* venom was fractionated on Hiload^TM^ 16/600 Superdex 75 prep grade column (1x120 ml) pre-equilibrated with 50 mM of Tris-Cl pH 7.4 using Äkta Purifier HPLC system, GE Healthcare (Uppsala, Sweden). Fractionation was carried out at a flow rate of 1 ml/min under isocratic conditions with the same buffer. Each eluted fraction was assayed for PLA_2_ and anticoagulant activity. P6 with highest anticoagulant and PLA_2_ activity was further subjected to cation exchange chromatography on Hiprep CM FF 16/10 (1.6x10 cm) using Äkta Purifier HPLC system. Elution was carried out at a flow rate of 2.25 ml/min with a linear gradient of 50 mM Tris-Cl, pH 7.4 containing 0.8 M NaCl. Ion exchange fraction, CM-II with highest PLA_2_ and anticoagulant activity was subjected to Rp-HPLC using Jupiter C_18_ column (3 μ, 4.6 x 250 mm, 300 Å). The column was pre-equilibrated with milli q containing 0.1% trifluoro acetic acid (TFA) and fractionated using 80% acetonitrile (MeCN) containing 0.1%TFA on Äkta Purifier HPLC system. The homogeneity of the purified protein (5 μg of the protein treated with 0.45 μl of β-mercaptoethanol) was validated on SDS-PAGE and stained using Pierce^TM^ silver staining kit, Life technologies, Thermo Fischer Scientific (MA, USA) [28]. This Rp-HPLC purified protein, named as daboxin P was used for characterization in the following experiments.

### Biophysical characterization

#### Determination of molecular mass

Molecular mass of daboxin P was determined by electrospray ionization mass spectrometry (ESI-MS) using LCQ fleet Ion Trap, Thermo Scientific (MA, USA). Ion spray voltage was set at 4.4 kV. For nebulization nitrogen gas was used. Solvent (50% MeCN containing 0.1% formic acid) was delivered into the system at a flow rate of 50 μl/min by Accela 600 pump. Spectra were recorded in positive mode with mass to charge (m/z) ranging from 800 to 2000. Analysis and deconvolution of the spectra was performed by Promas for Xcaliber.

#### Primary structure determination

N-terminal sequencing: N-terminal sequencing of the native purified protein was performed by automated Edman degradation process using Protein sequencer PPSQ 31A Shimadzu Asia Pacific, (Singapore). Briefly, 89 μg (6545.56 pmol) of the purified protein (dissolved in 100 μl of milli q water) was dried on PVDF membrane and loaded onto the sequencer. Sequencing was performed for 30 cycles or till the chromatogram was readable.

Pyridylethylation: Prior to chemical cleavage, daboxin P was reduced and alkylated according to the method developed by Joseph and colleagues [29,30]. Briefly, 1 mg of daboxin P was dissolved in 920 μl of denaturing buffer (6 M guanidine hydrochloride and 0.1 M Tris-Cl pH 8.5) and treated with β-mercaptoethanol (30 μl). N_2_ gas was purged over the reaction mixture to expel any dissolved oxygen and incubated at 37°C for 150 min. 50 μl of 4-vinyl pyridine (containing 100 ppm hydroquinone) was added and further incubated at 37°C for 150 min. The pyridylethylated sample was desalted by Rp-HPLC on Jupiter C_18_ column (4.6 x 250 mm, 3 μm, 300 Å) with a linear gradient of 80% MeCN containing 0.1% TFA at a flow rate of 0.8 ml/min. Elution was monitored at 215 and 280 nm.

Chemical cleavage: The chemical cleavage of pyridylethylated protein by BNPS-skatole and hydroxylamine hydrochloride were carried out according to the protocol developed by Crimmins and co-workers and Milner and colleagues respectively with minor modifications [31,32]. For cleavage with BNPS-skatole, 900 μg of the pyridylethylated protein was dissolved in 0.1% of TFA containing 6 M guanidine hydrochloride (pH 5.0). To this 1.5 mg/ml of BNPS skatole (dissolved in 100% acetic acid) was added and incubated at 37°C for 24 h. The reaction was stopped by addition of equal volume of milli q water. Centrifugation was carried out at 12,000 rpm for 20 min and supernatant was collected.

For cleavage with hydroxylamine hydrochloride, 1 mg of pyridylethylated protein was dissolved in denaturing buffer (6 M guanidine hydrochloride, 0.1 M Tris-Cl, pH 9.0, 2 M hydroxylamine hydrochloride and 0.2 M K_2_CO_3_) and incubated at 45°C for 4 h. After the cleavage experiments, the samples were subjected to Jupiter C_18_ column using a linear gradient of 5–50% of 80% MeCN containing 0.1% TFA. The mass of the fractions obtained were checked using ESI-MS (Accela LCQ fleet ion trap, Thermo scientific, U.S.A) for the cleaved and uncleaved peptide fragments. The cleaved fractions were lyophilized and reconstituted in 100 μl of milli q water and loaded onto PVDF membrane for Edman degradation using Automated Protein sequencer PPSQ 31A, Shimadzu.

Enzymatic cleavage/ Tandem mass spectrometry: Daboxin P (50 μg) was treated with 50 mM of ammonium bicarbonate, 1% Protease Max and 0.5 M dithiothreitol at 56°C for 20 min. For alkylation, 550 mM of iodoacetamide was added and incubated in dark for 15 min. Following this, trypsin (1 μg/μl in 50 mM acetic acid) in the presence of 1% Protease Max was added to the reaction and incubated at 37°C for 3 h. Reaction was stopped by 100%TFA. Centrifugation was carried out at 12,000 rpm for 10 min and the supernatant was subjected to Tandem mass spectrometry as described previously [11]. The MS/MS spectra obtained were analyzed using Proteome Discoverer 3.1 with Sequest program.

#### Sequence alignment and phylogenetic analysis

PLA_2_ enzymes having maximum sequence similarity with daboxin P were retrieved by blastp analysis from the NCBI database and aligned using multiple sequence alignment software DNAMAN 4.1.5.1 (Lynnon BioSoft). The phylogenetic relationship among the PLA_2_ enzymes exhibiting anticoagulant activity were determined by the Mega 5.05 software using neighbor joining method with a bootstrap of 1000 replicates [33]. The evolutionary distance among the sequences was calculated with p-distance.

#### Secondary structure determination

The secondary structure was analyzed by circular dichroism (CD) spectroscopy using Jasco Spectropolarimeter J-810 (Tokyo, Japan). Briefly, 200 μl of 0.4 mg/ml of daboxin P (dissolved in milli q water) was subjected to the spectropolarimeter. The spectrum of the native protein was recorded at far UV scan from 260–190 nm at a speed of 50 nm/min in quartz cuvette (0.1cm path length) at room temperature.

The effect of different pH (phosphate buffered saline at pH 3.0, 7.4 & 12) and temperature (from 25°C to 100°C) on structural conformation of daboxin P (0.4 mg/ml) was determined under the same experimental conditions as described above. Three scans were recorded for each experimental condition and subtracted from blank to obtain the final spectra of the protein.

### Biochemical and biological characterization

#### PLA_2_ activity

The PLA_2_ activity of daboxin P was assayed using the sPLA_2_ assay kit (Cayman, MI, USA) according to the manufacturer’s protocol using diheptanoylthio-phosphatidylcholine as substrate. The specific activity was expressed in micromoles of phosphatidylcholine hydrolyzed per min per mg of enzyme. Bee venom PLA_2_ enzyme (0.001 μg/μl) was considered as the positive control for the experiment. The results are mean ± standard deviation (SD) of three independent experiments.

#### Alkylation

The alkylation of His48 of daboxin P was performed as described by Maduwage and co-workers [34]. Briefly, 150 μg of daboxin P was treated with 15 μl of p-Bromophenacyl Bromide (pBpB) (100 μg dissolved in 30 μl of 100% ethanol). Equal amount of the protein was treated with 15 μl of ethanol as the vehicle control. The reaction mixtures were incubated at room temperature for 20 h and subsequently dialyzed in 20 mM of Tris-Cl, pH 7.4 at 4°C for 3 times. The dialyzed sample was lyophilized in a pre-conditioned lyophilizer (112N-G, Hahntech, South Korea) at –80°C. The lyophilized sample was reconstituted in appropriate buffer to carry out the desired assays. The extent of alkylation was verified by analyzing it using ESI-MS.

#### Cytotoxicity study

Cell culture: The HEK-293 and MCF-7 cell lines were grown in DMEM media enriched with 10% FBS and 1% antibiotic (Strep-Pen). The cells were grown on 96 well plate to a confluency of 70–80% at 37°C for 2–3 days in CO_2_ (5%) incubator (Eppendorf, Hamburg, Germany). Cell viability was assessed by trypan blue stain and the cells were quantified on a haemocytometer.

The cytotoxic activity of the crude venom of *Daboia r*. *russelii* and daboxin P were determined by the MTT (3-(4,5-dimethylthiazol-2-yl)-2,5-diphenyltetrazolium bromide) based colorimetric method [35]. Briefly, in two independent set of experiments, the HEK-293 and MCF-7 cells were plated on 96 well plate at a concentration of 1x10^5^ cells/well and incubated for 48 h at 37°C in CO_2_ (5%) incubator. Subsequently, the cells were incubated with different concentration of crude *Daboia r*. *russelii* venom and daboxin P for 24 h at 37°C in CO_2_ (5%) incubator. Cells treated with 0.9% NaCl was considered as the vehicle control. After incubation, the changes in morphological pattern (if any) of the treated cells were observed in an Inverted microscope (Axio Vert A1, Zeiss, Jena, Germany) at 10X magnification. 20 μl of MTT (5 mg/ml) was added and incubated at 37°C for 2 h. Following this, the formazan crystals formed by the viable cells were dissolved by 150 μl of MTT solution. The amount of formazan formed by the viable cells was quantified at 590 nm using MultiSkan Go spectrophotometer (Thermo Scientific, MA, USA). The percentage of cell viability was calculated by considering the cells without the venom treatment as 100% viable. The results are mean ± SD of three independent experiments.

#### *In vitro*-anticoagulant activities

The anticoagulant experiments were carried out with platelet poor human plasma (PPP) (whole blood centrifuged at 3000 rpm for 20 min at 16°C) and clot formation was monitored using Tulip Coastat-1 coaguloanalyser (Alto Santa Cruz, Goa, India). For each assay, clotting time of plasma with Tris-Cl buffer (20 mM, pH 7.4) was taken as the normal clotting time. The results are mean ± SD of three independent experiments.

Recalcification time (RT): Different concentrations of daboxin P (0.001, 0.01, 0.1, 1 μM) were pre-incubated with 150 μl of plasma at 37°C for 2 min [36]. Formation of plasma clot was initiated by addition of 100 μl of 50 mM CaCl_2_.

Activated partial thromboplastin time (APTT): APTT was determined using Liquecelin (APTT reagent) according to the manufacturer’s instructions. Briefly, different concentrations of the purified protein (0.01, 0.1, 1 μM) were pre-incubated with human plasma (50 μl) and APTT reagent (50 μl) for 3 min at 37°C. Plasma clot was formed by addition of 25 mM CaCl_2_ (50 μl).

Prothrombin time (PT): PT was measured using Uniplastin (PT reagent) according to the instructions of manufacturer. In brief, different concentrations of daboxin P were pre-incubated with 50 μl of human plasma at 37°C for 2 min. PT reagent (50 μl) was added to initiate the clot formation.

Stypven time: Briefly, in a reaction volume of 300 μl different concentrations of daboxin P were pre-incubated with human plasma (75 μl) for 3 min at 37°C [36]. To this 75 μl of RVV-X (10 ng/ml) was added and incubated for 2 min at the same temperature. Clotting was initiated by addition of 25 mM of CaCl_2_.

Thrombin time: To determine the thrombin time, different amounts of the protein (1, 3, 5 μg) were incubated with human plasma (50 μl) for 3 min at 37°C [36]. To this 50 μl of thrombin (10 u/ml) was added to initiate clot formation.

Fibrinogenolytic activity: Fibrinogenolytic activity was determined according to the method developed by Ouyang C and Teng CM [37]. In brief, different amounts of daboxin P were pre-incubated with 2 mg/ml fibrinogen (dissolved in 50 mM Tris-Cl, pH 7.5, 0.15 M NaCl) for 24 h at 37°C. For positive control, equal amount of fibrinogen was incubated with thrombin (3 μl of 10 units/ml) while for negative control fibrinogen with 50 mM Tris-Cl, pH 7.5 was incubated under the same experimental conditions. The formation of fibrinogen degradation products were analyzed on 12.5% glycine SDS-PAGE stained with Coomassie Brilliant Blue R-250.

#### *In-vivo* anticoagulant activity

The *in-vivo* anticoagulant activity of daboxin P was determined by the FeCl_3_-induced carotid artery thrombosis model with minor modifications [38–40]. Briefly, C57BL/6 male mice (9–11 weeks old, 24–28 g) were anesthetized with ketamine (75 mg/kg) and medetomidine (1 mg/kg) by intraperitoneal injection (i.p.). Daboxin P (10 mg/kg) was injected into the mice (n = 6) via its tail vein. Following this, the right carotid artery was exposed using blunt dissection and a doppler flow probe (Model MA0.5VB, Transonic System Inc., Ithaca, NY, USA) connected to a perivascular flow module (TS420, Transonic System Inc., Ithaca, NY, USA) was then attached to the carotid artery to monitor blood flow. A 2x2 mm piece of filter paper (soaked in 10% FeCl_3_ solution) was placed on the surface of the carotid artery for 3 min to initiate the thrombus formation. Saline treated mice (n = 5) were considered as the negative control for the experiment. Time-to-occlusion (TTO) is defined as the time taken for the blood flow to reach zero after the application of FeCl_3_. Maximum measurement time was considered for 60 min after the application of FeCl_3_. TTO was recorded as 60 min, if no occlusion occurred by this time. The animal experiments were approved by Institutional Animal Care and Use Committee, National University of Singapore

#### Serine protease specificity

The effect of daboxin P on the amidolytic activity of serine proteases were determined using specific chromogenic substrates. The activated serine proteases involved in the extrinsic and intrinsic tenase complex namely, FXIIa (60 nM), FXIa (0.125 nM), FIXa (333 nM), FXa (0.43 nM), FVIIa (10 nM), sTF (30 nM) were reconstituted in the activation buffer containing 50 mM Tris-Cl pH 7.4, 1 mg/ml of BSA, 140 mM NaCl and 5 mM CaCl_2_ [41]. The respective chromogenic substrates, S-2302 (1 mM), S-2366 (1 mM), spectrozyme (0.4 mM), S-2765 (0.65 mM) and S-2288 (500 μM) were reconstituted in milli q water to the desired working concentration. Briefly, to a reaction volume of 200 μl, each of the serine proteases was pre-incubated with different concentration of daboxin P (0.01, 0.1 and 1 μM) for 5 min. Subsequently, respective chromogenic substrates were added to trigger the amidolytic reaction. For each assay, the rate of p-nitroaniline formation upon hydrolysis of the substrate by the specific enzyme was quantified at 405 nm using UV-Vis MultiSkan GO spectrophotometer. The hydrolysis of substrate in the absence of daboxin P was considered as 100%.

#### Reconstitution of the tenase complexes

Extrinsic tenase complex (ETC) with and without phospholipids: The extrinsic tenase complex was reconstituted under *in-vitro* conditions in the presence of FVIIa (5 nM) and Innovin (10X) (tissue factor, thromboplastin and Ca^+2^ ions) with 67 μM of phospholipid vesicles (DOPC:DOPS 7:3) in the activation buffer. The reaction mixture was incubated at 37°C for 15 min [20]. Similarly, the complex was formed without phospholipids with 20 nM of FVIIa and soluble tissue factor, sTF (60 nM) and incubated for 15 min at 37°C.To both the experiments, different concentrations of daboxin P (0.01, 0.1, 1 and 3 μM) were added and incubated for 15 min. Following this, the reactions were incubated with FX (30 nM) for 15 min. Both the experiments were stopped by addition of quenching buffer (50 mM Tris-Cl pH 7.4, 1 mg/ml of BSA, 140 mM NaCl and 50 mM EDTA). The amount of FXa generated was determined by addition of 500 μM of S-2222 and the rate of hydrolysis was quantified at 405 nm using UV-Vis MultiSkan GO spectrophotometer.

Intrinsic tenase complex with and without phospholipids (ITC): Reconstitution of the intrinsic tenase complex under *in-vitro* conditions was carried out in the presence of phospholipids (67 μM) in activation buffer with FVIIIa, FIXa, FX and Ca^+2^ ions at 37°C [42,43]. Briefly, 3 nM of FVIII was incubated with 500 pM of thrombin for 10 min to activate FVIII. Thrombin was then deactivated by 10 nM of variegin, a thombin inhibitor from *Amblyomma variegatum* [44]. After incubation of the reaction mixture with 1 nM of FIXa for 10 min different concentrations of daboxin P (0.01, 0.1, 1 and 3 μM) were added and incubated for 15 min. Following this, 25 nM of FX was added and incubated for 15 min. Similarly, the complex was formed devoid of the phospholipid blend [42] with FVIII (40 nM) and thrombin (4 nM) and incubated for 10 min. Thrombin was deactivated with 40 nM of variegin. To this 10 nM of FIXa was added and incubated for 10 min. Then, different concentrations of daboxin P (0.01, 0.1, 1 and 3 μM) were incubated for 15 min. Finally, 1 μM of FX was added and incubated for 15 min. The reactions in both the experiments were terminated by addition of the quenching buffer. The amount of FXa generated was quantified by the hydrolysis of the chromogenic substrate S-2222 (500 μM) at 405 nm using UV-Vis MultiSkan GO spectrophotometer.

For determining the inhibitory concentration (IC50) of daboxin P on extrinsic and intrinsic tenase complex, different concentration of daboxin P were pre-incubated with each of the reconstituted complexes described above in the presence of phospholipids in the activation buffer.

Prothrombinase complex: Prothrombinase complex was reconstituted in the presence of phospholipid (67 μM) with FXa, FVa, and Ca^+2^ ions under *in-vitro* conditions at 37°C [19]. Briefly, 10 pM of FXa was pre-incubated with 1 nM of FVa for 15 min. To this, different concentrations of daboxin P (0.01, 0.1, 1 and 3 μM) were added and incubated for 15 min. Thereafter, prothrombin (12.5 nM) was added and the reaction mixture was incubated for another 15 min. In another set of experiment, FXa (10 pM) was pre-incubated with different concentrations of daboxin P followed by incubation with FVa (1 nM) and prothrombin (12.5 nM) for 15 min each. For both the experiments, the reactions were stopped with quenching buffer. The amount of thrombin generated by the complex was determined by the rate of hydrolysis of the chromogenic substrate S-2238 (250 μM) at 405 nm using UV-Vis MultiSkan GO spectrophotometer.

#### Fluorescence emission spectroscopy

Interaction of daboxin P with the coagulation factor FX and FXa were analyzed using fluorescence emission spectrophotometer (LS 55, Perkin Elmer, Palo Alto, CA). In brief, 1 μM of daboxin P (dissolved in 20 mM Tris-Cl, pH 7.4) was incubated with either 0.05 μM of FX or 0.1 μM of FXa (reconstituted in the same buffer) for different time intervals (10 min & 20 min) at room temperature in the presence and absence of 5 mM CaCl_2_. The change in the fluorescence emission spectra of the individual components and the mixtures were measured over a wavelength of 200 to 500 nm with an excitation wavelength of 280 nm at room temperature using a quartz cuvette (1 cm path length).

#### Affinity chromatography

The association of daboxin P with FX and FXa were evaluated by affinity column chromatography using CNBr activated sepharose® 4B (Sigma, Aldrich). Briefly, 40 μg of daboxin P was covalently linked to 0.1 g of CNBr activated matrix (swollen overnight in ice cold 1 mM HCl) in the presence of coupling buffer containing 0.1 M NaHCO_3_ and 0.5 M NaCl, pH 8.3. Subsequently, the unbound protein molecules of daboxin P were washed away with the same buffer. To avoid non-specific binding, the immobilized matrix was treated with 0.2 M glycine for 2 h with gentle shaking at room temperature. The blocking solution was removed by an alternative wash with acidic (0.1 M acetate buffer, pH 4.0) and basic buffer (coupling buffer) for 5 times. FX or FXa (8 μg) were added to the daboxin P immobilized matrix and incubated for 1 h at room temperature with mild shaking. The unbound FX or FXa molecules were washed away with the same buffer. Elution of FX or FXa was carried out with 20 mM HCl containing increasing molar concentrations of NaCl (0.5, 1, 1.5 and 3 M). The flow through and eluents were analyzed on 12.5% glycine SDS-PAGE under non-reducing condition and visualized after staining with Pierce^TM^ silver staining kit.

#### Modelling and molecular docking studies

Three dimensional (3D) molecular modelling: For the *in-silico* 3D modelling of daboxin P, online server I-TASSER was employed [45–47]. This server predicts the secondary conformation of a given protein by profile-profile alignment (PPA) threading techniques. The model with maximum C-score and TM score was selected for generation of ribbon model using DS ViewerPro 5.0 (Accelrys Inc.) software. Superimposition of the predicted model with the crystal structure of ammodytoxin A, AtxA (Pdb 3G8G), was carried out using DS ViewerPro 5.0 software.

Molecular docking: The equilibrated model of daboxin P was docked with FXa (PDB: 2BOH) using the online web server, PatchDock [48,49]. This server uses the geometry-based docking algorithm to find optimum candidate solutions and the RMSD (root mean square deviation) clustering to remove redundant solutions [49]. Each solution was given score based on the geometric fit as well as atomic desolvation energy [50]. In the present analysis, a default RMSD value of 4 Å was considered for clustering solutions. The ribbon model of the docked complex of daboxin P and FXa was generated using DS ViewerPro 5.0 (Accelrys Inc.).

Identification of interface residues and hot spot residues: The daboxin P-FXa docked model with maximum surface contact area and minimum free energy generated by PatchDock server was selected for evaluating the interface residues using PDBsum server [51,52]. The interface residues of a protein are those residues whose contact distances from the interacting protein partner are less than 6 Å [53].

Contact map analysis: The interaction between the chains of FXa and daboxin P were also analyzed using CMA (Contact Map Analysis), one of the servers of the online software SPACE (**S**tructure **P**rediction and **A**nalysis based on **C**omplementarity with **E**nvironment) [54]. The server analyses the contact area between the residues of two protein chains or within a single chain of a given PDB file based on shape and chemical complementarity. It displays residue to residue contacts for a pair of amino acid residues involved in the interaction in the form of contact map. Contact maps provide more concrete demonstration of protein structure than its 3D atomic coordinates. For investigating the residue-residue contacts between daboxin P and FXa, a contact area threshold above 8Å^2^ has been considered for the present analysis.

## Results

### Purification of daboxin P, an anticoagulant PLA_2_ enzyme

**Fig 1:**
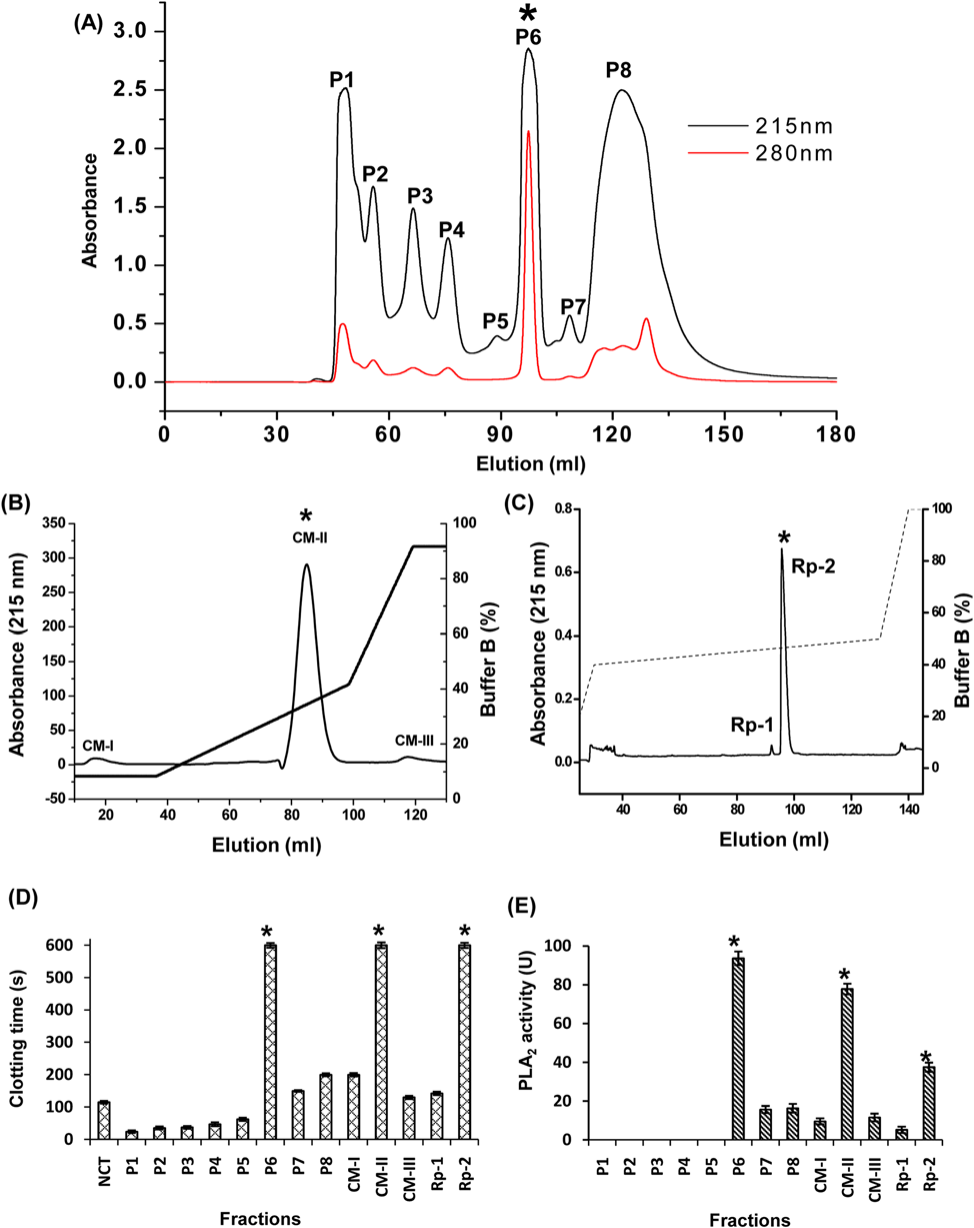
Purification of the major protein from the crude venom of Indian *Daboia r*. *russelii*. **(A):** Size exclusion chromatography of crude *D*. *r*. *russelii* venom. 20 mg of crude venom was dissolved in 50 mM of Tris–Cl pH 7.4 and fractionated on Hiload 16/600 superdex 75 preparative grade column pre-equilibrated with the same buffer. Fractions were eluted at a flow rate of 1 ml/min and monitored at 215 & 280 nm. For each, 1 ml fractions were collected and peaks were pooled (P1 to P8). **(B):** Ion exchange chromatography profile of P6: The gel filtration peak, P6 was loaded onto CM FF 16/10, a weak cation exchanger column pre-equilibrated with 50 mM of Tris-Cl pH 7.4. Fractionation was carried out at a flow rate of 2.25 ml/min and eluted with a linear gradient of the same buffer containing 0.8 M NaCl and monitored at 215 nm. **(C):** Rp-HPLC profile of CM-II: Ion exchange fraction CM-II was loaded on Jupiter C_18_ column pre-equilibrated with buffer A (0.1% TFA). Fractionation was carried out at a flow rate of 0.8 ml/min with a linear gradient of buffer B (80% MeCN+0.1% TFA) and monitored at 215 nm. **(D):** Recalcification time of the fractions obtained from the chromatographic steps. Clotting time of plasma in presence of Tris-Cl buffer (20 mM, pH 7.4) was considered as normal clotting time (NCT). 1 μg of each fraction was incubated with plasma for 2 min followed by addition of 50 mM CaCl_2_ to initiate clot formation which was monitored using Tulip Coastat-1 coagulo analyser. **(E):** PLA_2_ activity of the chromatographic fractions using sPLA_2_ assay kit. 0.01 μg of each fraction was used for screening PLA_2_ activity using diheptanoylthio-phosphatidylcholine as the substrate. The amount of substrate hydrolyzed was quantified at 414 nm for 10 min at room temperature. * indicates the peak of interest in each purification step.

**Fig 2:**
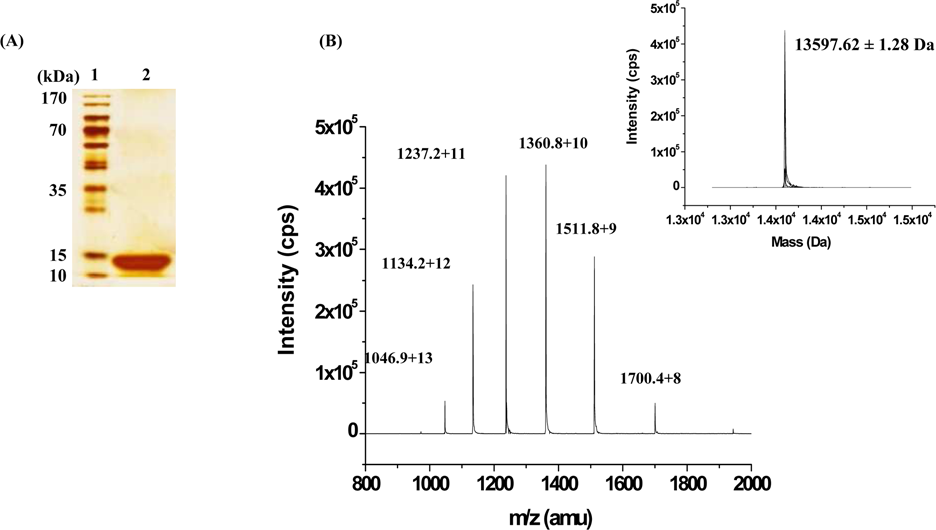
Homogeneity of the purified protein, daboxin P. **(A)** 12.5% glycine SDS-PAGE profile of the purified protein after silver staining. **Lane 1:** PageRuler^TM^ pre-stained protein marker (170–10 kDa). **Lane 2:** daboxin P after treatment with β-mercaptoethanol. **(B):** ESI-MS spectra. The spectra show a series of multiple charged ions corresponding to a homogenous peptide. **Inset:** Reconstructed mass of daboxin P (cps: counts per second, amu: atomic mass unit).

Fractionation of crude venom of *Daboia r*. *russelii* on size exclusion chromatography has resolved it into 8 prominent peaks (P1 to P8) (Fig 1A). Each individual peaks were screened for PLA_2_ and anticoagulant activity. Peaks, P1 to P5 were found to be devoid of PLA_2_ activity but exhibited procoagulant effect on human plasma while peaks P6 to P8 showed PLA_2_ activity and anticoagulant effect on human plasma (Fig 1D & 1E). Peak P6 which exhibited highest anticoagulant (>600 s) and PLA_2_ activity (93.69 μmol/min) was subjected to cation exchange chromatography. It resolved P6 into one major (CM-II) and two minor peaks (CM-I & CM-III) (Fig 1B). The major peak CM-II which showed highest anticoagulant (>600 s) and PLA_2_ activity (77.82 μmol/min) (Fig 1D & 1E), was further fractionated using Rp-HPLC, which resolved into one minor (Rp-1) and one major (Rp-2) peaks (Fig 1C). The homogeneity of the major peak, Rp-2 with highest PLA_2_ activity and anticoagulant activity was assessed by SDS-PAGE under reduced state and named as daboxin P (Fig 2A). The molecular mass of the protein was determined by ESI-MS which showed the presence of 6 mass to charge (m/z) ratio peaks ranging from +8 to +13 charges corresponding to a deconvoluted molecular mass of 13597.62 ± 1.28 Da (Fig 2B).

### Biophysical characterization

#### Sequencing

**Fig 3:**
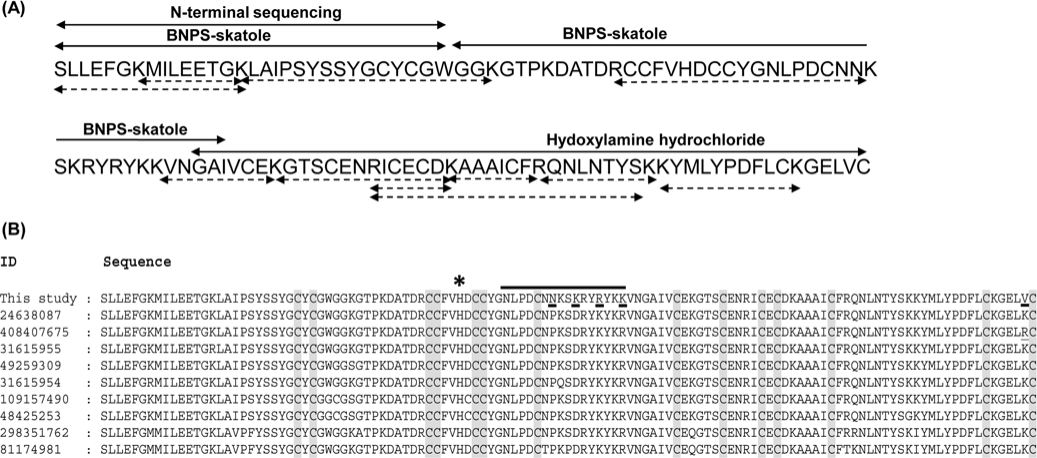
Primary structure of daboxin P. **(A):** Amino acid sequence was deciphered by Edman degradation sequencing and ESI-LC MS/MS. The peptide sequences obtained from N-terminal sequencing and chemical cleavage by BNPS-skatole and hydroxylamine hydrochloride are shown with two headed solid arrows whereas peptide sequences obtained after ESI-LC MS/MS of the tryptic digested fragments are indicated with two headed doted arrows. **(B):** Multiple sequence alignment of daboxin P with the PLA_2_ enzymes from different subspecies of *Daboia russelii* (**24638087:** *D*. *r*. *russelii*, **408407675:** *D*. *r*. *siamensis*, **31615955:** *D*. *r*. *pulchella*, **49259309:** *D*. *r*. *russelii*, **31615954:** *D r*.*pulchella*, **109157490:** *D*. *r*. *pulchella*, **48425253:** *D*. *r*. *pulchella*, **298351762:** *D*. *r*. *russelii*, **81174981:** *D*. *r*. *russelii*). The conserved cys residues are highlighted in grey and the amino acid substitutions in daboxin P are underlined. * indicates the His residue at the active site. The predicted anticoagulant region is highlighted with a solid black line.

The first 30 residues were determined by N-terminal sequencing (Fig 3A). The cleavage of pyridylethylated daboxin P with BNPS-skatole yielded two peptide fragments of molecular mass 11.7 kDa and 3.6 kDa (data not shown) while cleavage with hydroxylamine hydrochloride resulted into two peptide fragments of molecular mass 6.6 kDa and 8.6 kDa (data not shown). Sequencing of these peptides by Automated Edman degradation revealed the rest of the amino acid residues (Fig 3A). On the other hand from tandem mass spectrometry, eleven peptide fragments of daboxin P were obtained (Table A in S1 File). These peptide fragments obtained by chemical and enzymatic cleavage were assembled and overlapped to decipher the complete sequence of daboxin P (Fig 3A). The homology of the protein sequence was analyzed by multiple sequence alignment with PLA_2_ enzymes using online blastp algorithm as represented in Fig 3B.

#### Sequence analysis

The calculated molecular mass of daboxin P was found to be 13596.70 Da which is in agreement with the observed mass of 13597.62 ± 1.28 Da from ESI-MS. It has 121 amino acid with 14 cysteine residues which should correspond to 7 disulphide bonds, a characteristic feature of group IIA viper PLA_2_ enzymes [55,56]. It has a theoretical pI of 8.5, calculated by online software ExPASy ProtParam [57]. At the active site, histidine residue was observed followed by an aspartate residue, a common trait for Ca^+2^ dependent catalytic PLA_2_ enzymes [58]. At the predicted anticoagulant region (53NLPDCNNKSKRYRYKK68) positively charged amino acid residues (Lys and Arg) were observed which propose it to be a strong anticoagulant PLA_2_ enzyme.

Homology search by blastp shows its similarity to VRV-PL-VIIIa (96%) isolated from *Daboia r*. *russelii* at position 59^th^ (Asn), 62^nd^ (Lys), 65^th^ (Arg), 68^th^ (lys) and 120^th^ (Val) (Fig 3B) [59].

#### Phylogenetic relationship

Daboxin P showed close phylogenetic relation with the reported anticoagulant PLA_2_ enzymes which are basic, like vurtoxin which is a strong anticoagulant enzyme reported from *Vipera renardi* and FXa binding enzymes like DPLA2 from *Daboia russelii pulchella*, AtxA and AtxC from *V*. *a*. *ammodytes* with a bootstrap value of 99 [59–61] (Figure A in S1 File).

#### Secondary structure determination

**Fig 4:**
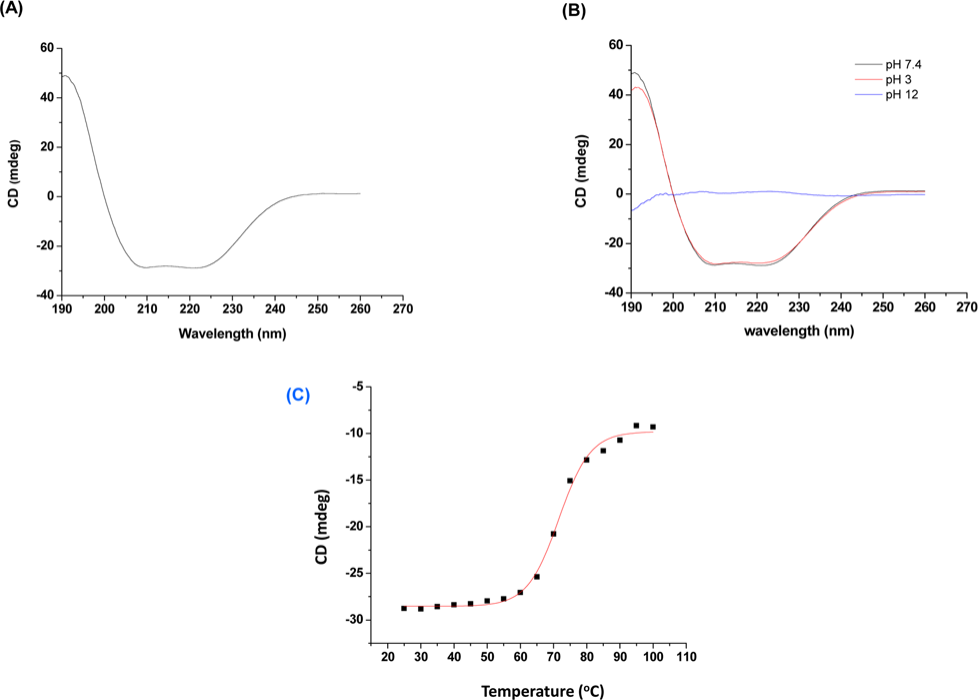
Far-UV circular dichroism (CD) spectra of daboxin P (0.4 mg/ml). (**A):** in milli q water at 25°C, **(B):** at different pH (3.0, 7.4 & 12) at 25°C. **(C)** Melting curve of daboxin P (dissolved in milli q water) at 222 nm considering temperature as a function. The curve was plotted using sigmoidal curve fit and Tm value was determined by Boltzman equation using Origin (OriginLab).

The far UV CD spectrum has shown negative minima at 222 nm and 208 nm and positive maxima at 190 nm, typical for α-helical pattern of PLA_2_ enzymes (Fig 4A). The percentage of secondary structure was determined by online software K2D3 which calculated 42.73% of α-helix and 12.36% of β-sheet in daboxin P [62,63].

The CD spectrum at acidic pH (3.0) and neutral pH (7.4) showed stable structural conformation. However, the structure was distorted completely at alkaline pH (12.0) with residual α-helix of 5.51% & β-sheet of 23.75% (Fig 4B). On the other hand, its secondary structure showed a Tm (melting temperature) value of 71.59 ± 0.4°C when scanned over a range of temperature (Fig 4C).

### Functional characterization

#### Catalytic activity

**Fig 5:**
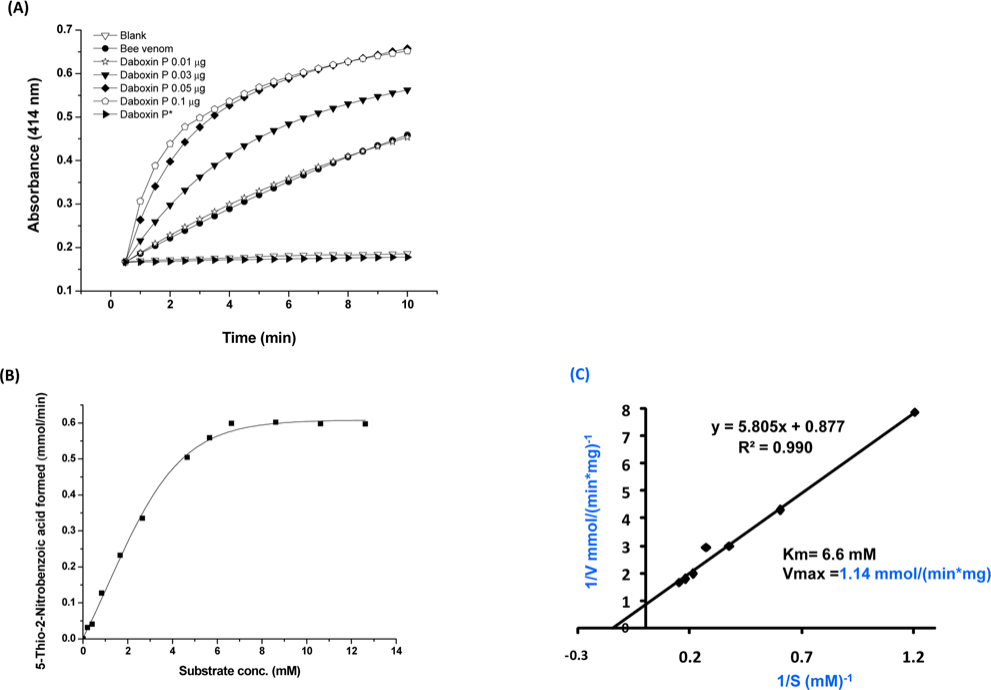
Phospholipase A_2_ activity of daboxin P. **(A)** Progress curve of diheptanoyl thio-PC cleavage by daboxin P, bee venom PLA_2_ enzyme and histidine modified daboxin P* at 414 nm. **(B)** Michaelis-Menten’s curve for sPLA_2_ assay. **(C)** The Lineweaver-Burk plot of daboxin P for determination of Km and Vmax.

Daboxin P exhibited catalytic activity on diheptanoylthio-phosphatidylcholine (PC) in a dose dependent manner (Fig 5A). The Michaelis Menten plot with different substrate concentration showed a hyperbolic curve for enzyme activity and the Km (6.6 mM) and Vmax (1.14 mmol/(min*mg)) were determined using Lineweaver Burk-plot (Fig 5B & 5C). Alkylation with pBpB has led to the complete loss of its enzymatic activity signifying the crucial role of histidine residue in catalysis (Fig 5A).

#### Cytotoxicity

**Fig 6:**
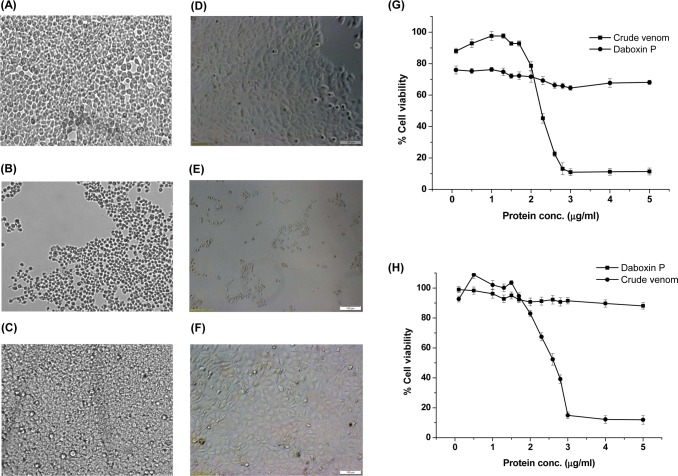
Cytotoxic effect of crude *Daboia r*. *russelii* venom and daboxin P. Microscopic images were photographed at 10X magnification under Inverted microscope (Axio Vert A1., Zeiss) after treatment with venom samples for 24 h **(A):** HEK-293 cells treated with 0.9% NaCl were considered as negative control **(B):** HEK-293 cells treated with crude *Daboia r*. *russelii* venom (5 μg/ml) **(C):** HEK-293 cells treated with daboxin P (5 μg/ml) **(D):** MCF-7 cells treated with 0.9% NaCl were considered as negative control **(E):** MCF-7 cells treated with crude *Daboia r*. *russelii* venom (5 μg/ml) **(F):** MCF-7 cells treated with daboxin P (5 μg/ml). Percentage cell viability **(G):** HEK-293; **(H):** MCF-7 after treatment with crude venom and daboxin P using MTT based colorimetric assay. Percentage cell viability was calculated by considering the cells without venom treatment as 100% viable. The results are mean ± SD of three independent experiments.

The crude venom of *Daboia r*. *russelii* has shown prominent cytotoxic effect on HEK-293 and MCF-7 cells in a dose dependent manner (Fig 6). At a concentration of 2.8 and 3 μg/ml of crude venom, only 13.09% of HEK-293and 14.97% of MCF-7 cells respectively (Fig 6B, 6E, 6G and 6H) remained viable while the same concentrations of daboxin P did not show any cytotoxic effect, suggesting that the cytotoxic effect of the crude venom is not due to this protein (Fig 6C and 6F–6H).

#### *In-vitro* anticoagulant activity

**Fig 7:**
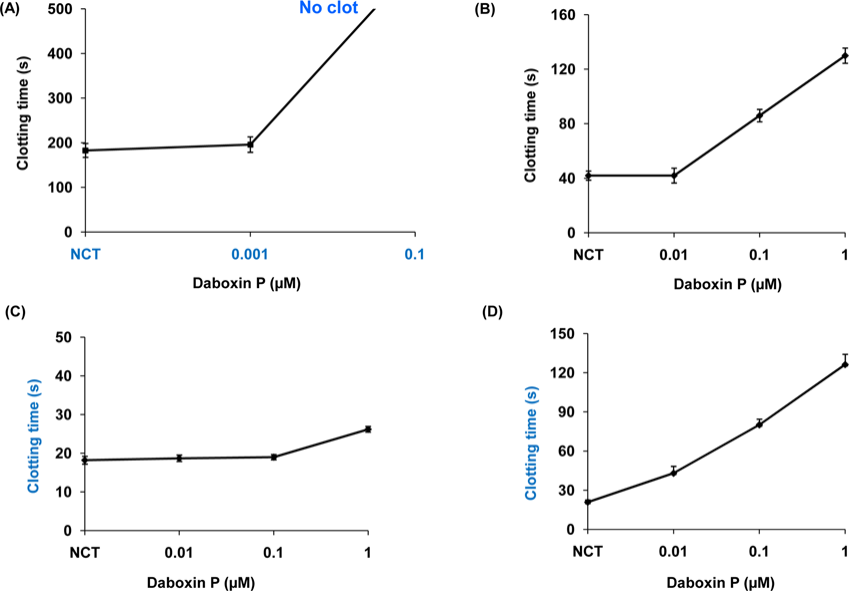
Anticoagulant activities of daboxin P on platelet poor human plasma (PPP). **(A):** Recalcification time, different concentrations of daboxin P (0.001, 0.01, 0.1) were pre-incubated with 150 μl of plasma at 37°C for 2 min. 100 μl of 50 mM CaCl_2_ was added to initiate clot formation. **(B):** Activated partial thromboplastin time, daboxin P (0.01, 0.1 & 1 μM) was pre-incubated with 50 μl of plasma and 50 μl APTT reagent (Liquecelin) for 3 min at 37°C. 50 μl of 25 mM CaCl_2_ was added to form clot. **(C):** Prothrombin time, different concentrations of daboxin P (0.01, 0.1 & 1 μM) were pre-incubated with 50 μl of plasma at 37°C for 2 min. 50 μl of PT reagent (Uniplastin) was added to initiate the clot formation. **(D):** Stypven time, daboxin P (0.01, 0.1 & 1 μM) was pre-incubated with 75 μl plasma for 3 min at 37°C. 75 μl RVV-X (10 ng/ml) was added and incubated for 2 min. 25 mM of CaCl_2_ was added to initiate clot formation. For all the experiments, the clot formation was monitored using Tulip Coastat-1 coagulo analyser and the time taken for clot formation in the presence of Tris-Cl buffer (20 mM, pH 7.4) was considered as normal clotting time (NCT). The results are mean ± SD of three independent experiments.

Daboxin P exhibited anticoagulant effect on the various coagulation assays in a dose dependent manner (Fig 7). At a concentration of 0.01 μM, it prolonged the recalcification time beyond 600 s indicating it to be a strong anticoagulant enzyme (Fig 7A). 1 μM of the protein delayed activated partial thromboplastin time up to 130 s but did not exhibit prominent effect on prothrombin time (Fig 7B & 7C). Daboxin P exhibited anticoagulant effect on stypven time in a dose dependent manner displaying its inhibitory effect on FX (Fig 7D). Nevertheless, it did not show any inhibitory effect on thrombin time and was devoid of fibrinogenolytic activity (Figure B i & ii in S1 File). This suggests that daboxin P might target a component/coagulation factor upstream of the common pathway.

#### *In-vivo* anticoagulant activity

**Fig 8:**
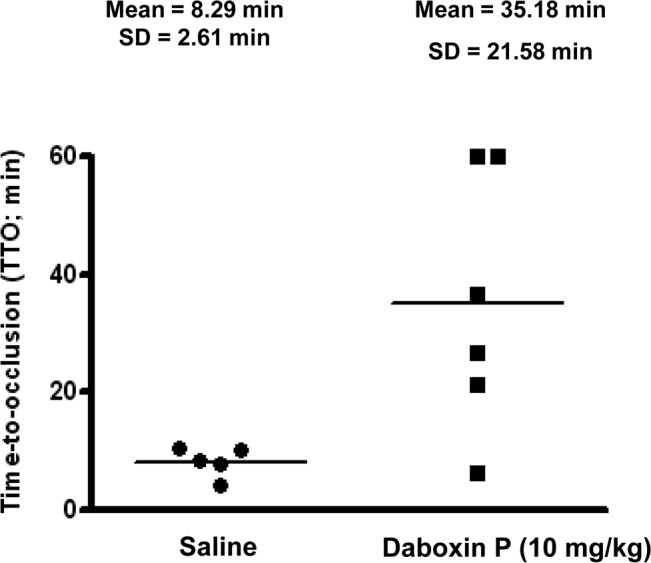
Effect of daboxin P on the time to occlusion (TTO) in FeCl_3_ induced carotid artery thrombosis in mice. C57BL/6 male mice anesthetized with ketamine (75 mg/kg) and medetomidine (1 mg/kg) (i.p) were injected (i.p.) with daboxin P (10 mg/kg) in tail vein. Saline treated mice were considered as negative control. Each data-point represents the time-to-occlusion (TTO) of a single mouse. Maximum experimental time was considered for 60 min after FeCl_3_ induction.

Daboxin P exhibited anticoagulant effect in the carotid artery of mouse treated with FeCl_3_. Mice administered with saline had a time-to-occlusion within 10 min. However, daboxin P administration resulted in ~4-fold increase (35.18 ± 21.58 min) in the time-to-occlusion in comparison to the saline treated mice (8.29 ± 2.61 min) (Fig 8). This result was in accordance with the *in-vitro* anticoagulant assays on human plasma and highlighted the anticoagulant property of daboxin P under *in-vivo* condition.

#### Serine protease specificity

**Fig 9:**
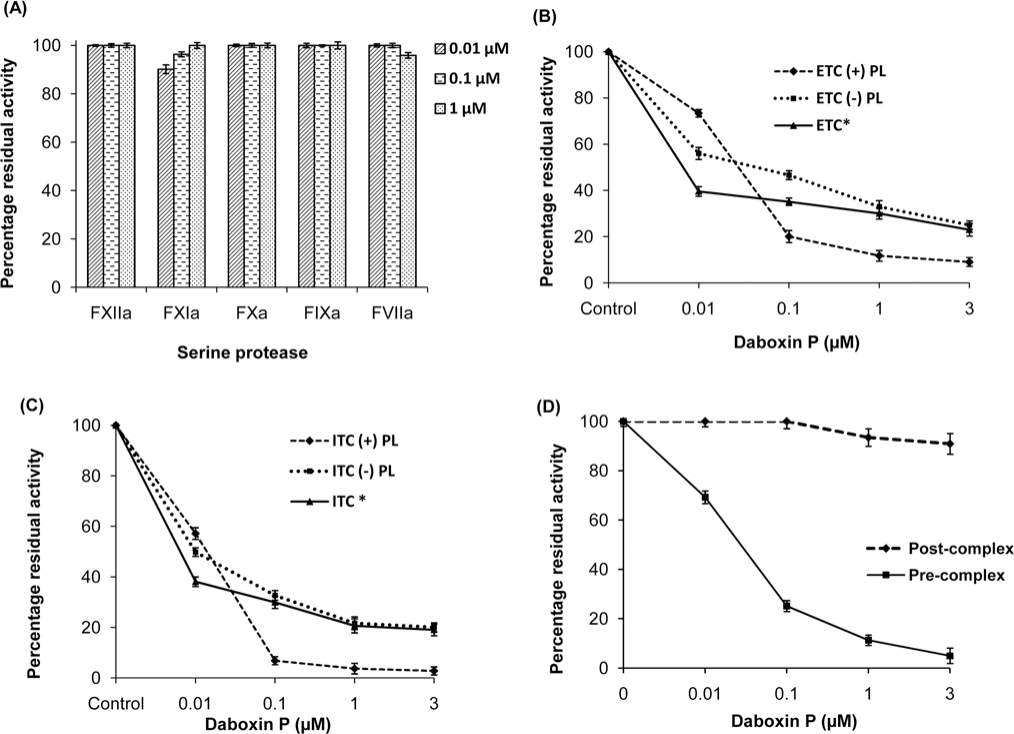
Percentage residual amidolytic activity of various serine proteases and complexes pre-incubated with daboxin P. **(A):** Residual activity of FXIIa, FXIa, FXa, FIXa and FVIIa; **(B):** Activity of extrinsic tenase complex (ETC) **(C):** intrinsic tenase complex (ITC), in the presence or absence of phospholipid and alkylated daboxin P (indicated by *). **(D):** Residual activity of prothrombinase complex. Daboxin P was either pre-incubated with FXa followed by addition of FVa (pre-complex) or after reconstitution of FXa-FVa complex (post-complex). The rate of hydrolysis of respective chromogenic substrates for all the assays was measured at 405 nm using Multiskan Go spectrophotometer. Activity of the serine protease/complex without daboxin P was considered as 100%. The results are mean ± SD of three independent experiments.

**Fig 10:**
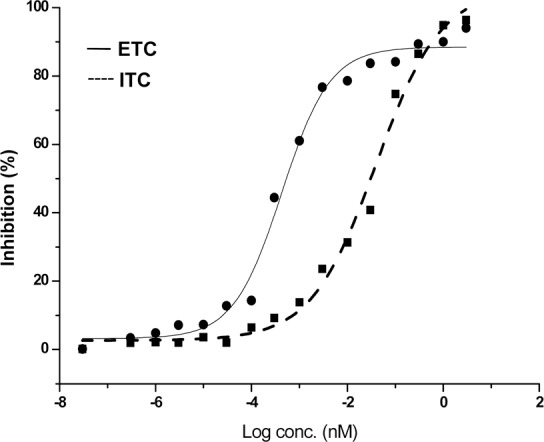
Inhibition curve (IC50) of daboxin P for the extrinsic and intrinsic tenase complex. Different concentrations of daboxin P (1x10^-6^, 1x10^-5^, 1x10^-4^, 1x10^-3^, 1x10^-2^, 1x10^-1^, 1, 3x10^-6^, 3x10^-5^, 3x10^-4^, 3x10^-3^, 3x10^-2^, 3x10^-1^ and 3 μM) were pre-incubated with reconstituted tenase complexes as described above. The IC_50_ was calculated by fitting the points by non-linear curve fit using Origin (OriginLab, Northampton, MA).

Screening for inhibitory effect of daboxin P on the amidolytic activity of various serine proteases involved in the extrinsic (FVIIa), intrinsic (FXIIa, FXIa, FIXa) and common (FXa) pathway revealed that it did not inhibit any of these serine proteases when assayed using respective synthetic chromogenic substrates (Fig 9A). However, it exerted inhibitory effect on both the extrinsic (ETC) (IC50 = 0.43 nM) and intrinsic (ITC) (IC50 = 39.20 nM) tenase complexes causing hindrance in the activation of FX to FXa (Fig 10). The residual activity of ETC and ITC was observed to be 9.05% ± 1.9 and 2.8% ± 1.6 respectively upon treatment with 3 μM of daboxin P (Fig 9B & 9C). Further, when the tenase complexes were reconstituted without phospholipid, the residual activity of ETC and ITC was found to be 25% ± 2.7 and 20.05% ± 2.3 respectively. Moreover, when the tenase complexes were treated with alkylated daboxin P (3 μM), only 23% ± 1.8 (ETC) and 19.03% ± 1.8 (ITC) of residual activity remained which is comparable to the residual activity of both the complexes reconstituted without phospholipids (Fig 9B & 9C). Interestingly, the thrombin formation by the prothrombinase complex was unaffected when daboxin P was added after the formation of the FXa-FVa complex. However, only 11.28% of residual activity remained when it was pre-incubated with FXa followed by addition of FVa for the formation of prothrombinase complex (Fig 9D).

#### Fluorescence emission spectroscopy

**Fig 11:**
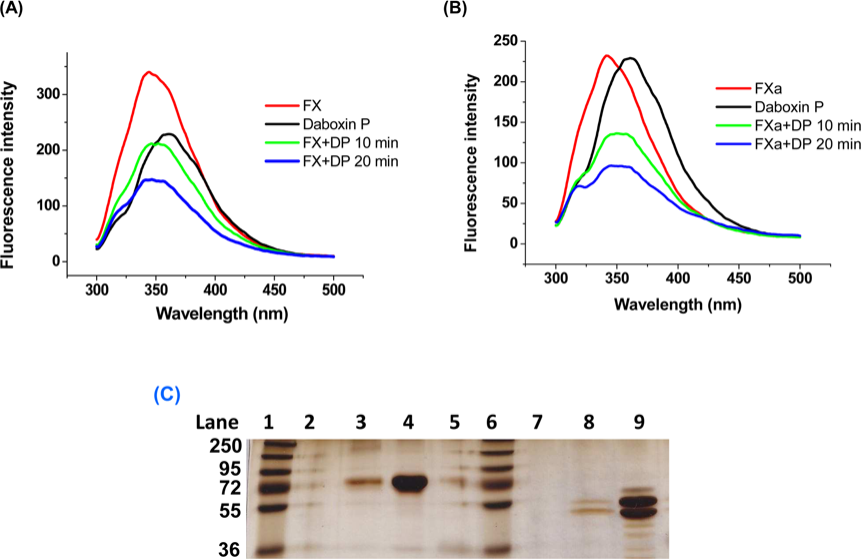
Interaction of daboxin P with FX and FXa. **(A):** Fluorescence emission spectra of daboxin P, FX, and the complex (daboxin P + FX). **(B)** Fluorescence emission spectra of daboxin P, FXa, and the complex (daboxin P + FXa). 1 μM of daboxin P (DP) was pre-incubated with either 0.05 μM of FX or 0.1 μM of FXa for different time intervals (10 min & 20 min) at room temperature. The emission spectra of the individual proteins and the complexes were measured from 200 to 500 nm with an excitation wavelength of 280 nm using quartz cuvette (1 cm path length). **(C):** Electrophoretic profile of the flow through and elute obtained after affinity column chromatography. **Lane 1**: PageRuler^TM^ Plus pre-stained protein ladder (250–10 kDa), **Lane 2:** flow through (FX) **Lane 3:** FX after elution with 1.0 M NaCl, **Lane 4:** control (FX); Lane 5: blank; **Lane 6:** PageRuler^TM^ Plus pre-stained protein ladder (250–10 kDa), **Lane 7:** flow through (FXa); **Lane 8:** FXa after elution with 1.0 M NaCl, **Lane 9:** control (FXa).

The fluorescence emission spectrum of FX and FXa was quenched by daboxin P with increasing incubation time (Fig 11A & 11B). The presence or absence of Ca^+2^ ions did not show any effect on the quenching spectra for both FX and FXa when treated with daboxin P (data not shown).

#### Affinity column chromatography

The electrophoretic profile of the flow through for both FX and FXa did not show any protein bands suggesting the binding of these proteins to the daboxin P immobilized CNBr matrix. Nevertheless, the eluent at 1.0 M NaCl showed prominent protein bands for both the serine proteases suggesting the disturbance of the protein-protein interaction at high salt concentration (Fig 11C).

#### Molecular modelling and protein-protein docking

The predicted 3D model of daboxin P has shown structural similarity to the crystal structure of AtxA (PDB: 3G8G) with a RMSD value of 0.9 Å which validates the predicted model (Figure C i in S1 File). The conserved critical regions and amino acid residues in daboxin P are shown in Figure C ii in S1 File.

**Fig 12:**
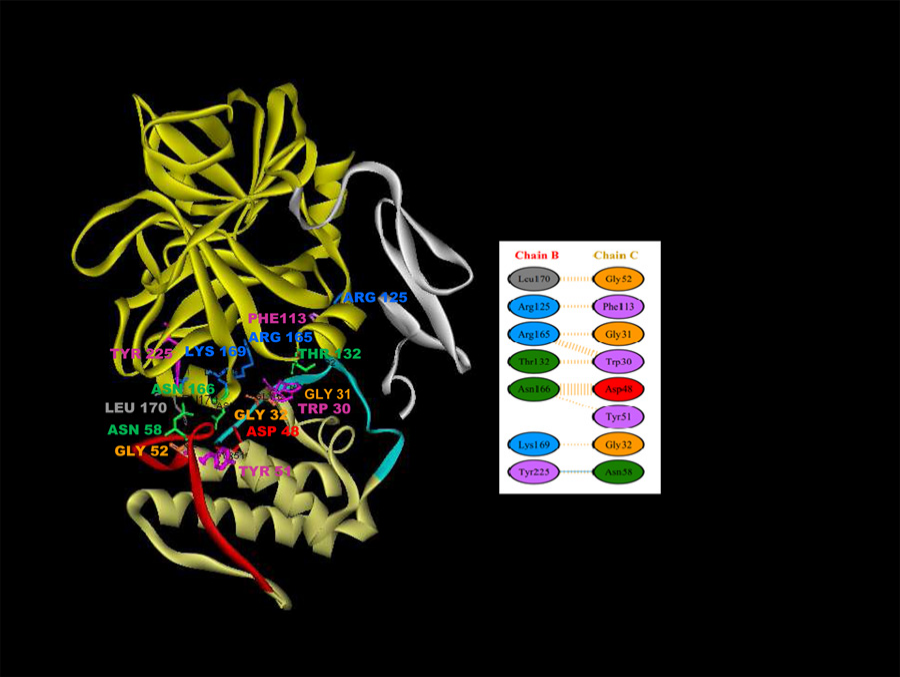
3D ribbon model of the docked complex of FXa and daboxin P. The interface surface residues involved in the interaction were predicted by PDBsum. The Ca^+2^ binding loop (Trp30, Gly31, Gly32); helix C (Asp48, Tyr51, Gly52); anticoagulant region (Asn58) and C-terminal region (Phe113) of daboxin P interact with the heavy chain of FXa (Thr132, Arg165, Lys169, Asn166, Leu170, Tyr225 and Arg125) are represented in scaled ball and stick. **Inset:** Diagram illustrating the interaction of the seven residues of chain B (heavy chain of FXa) with eight residues of chain C (daboxin P) as predicted by PDBsum server. Orange line denotes non-bonded contacts and blue line denote hydrogen bond.

The best protein-protein docked model of daboxin P and FXa chosen in terms of minimum free energy and maximum surface contact area, was obtained from PatchDock web server (Fig 12). Daboxin P interacts with the heavy chain (B-Chain) of FXa with a geometric shape complementarity score of 12234. Geometric scoring refers to good molecular shape complementarity between the docked chains due to optimal fit with wide interface areas and lesser steric clashes [49]. The approximate interface area of the complex was found to be 1837 Å^2^ with atomic contact energy (ACE) of 472.62 kcal/mol.

The interface and possible interacting residues across the interface of the complex were predicted by PDBsum. The total number of interface residues in protein-protein complex was found to be 31 and the interface area for each chain involved in the interaction was observed to be more than ~500 Å^2^. The docked complex was stabilized by molecular interactions like hydrogen bonding and non- bonded contacts. The interface amino acid residues of FXa (chain B) and daboxin P (chain C) involved in the interaction are shown in Fig 12 inset and the interface plot statistics is summarized in Table B in S1 File.

The docked complex shows interaction of daboxin P with the heavy chain (Chain B) of FXa at eight critical regions some of which are reported to be crucial for FVa binding [64]. The daboxin P residues Trp30, Gly31, Gly32 of the Ca^+2^ binding loop; Asp48, Tyr51, Gly52 of helix C; Asn58 of the anticoagulant region and Phe113 of C-terminal region interact with residues Thr132, Arg165, Lys169, Asn166, Leu170, Tyr225 and Arg125 of heavy chain of FXa (Table 1).

**Table 1:** Critical residues of daboxin P and FXa involved in interaction based on PDBsum analysis.

Daboxin P	FXa
Trp30, Gly31, Gly32 (Ca^+2^ binding loop)	Thr132, Arg165, Lys169 (Heavy chain)
Asp48, Tyr51, Gly52 (Helix C)	Asn166,Lys169,Leu170 (Heavy chain)
Asn58 (Anticoagulant region)	Tyr225(Heavy chain)
Phe113 (C-terminal region)	Arg125(Heavy chain)

**Fig 13:**
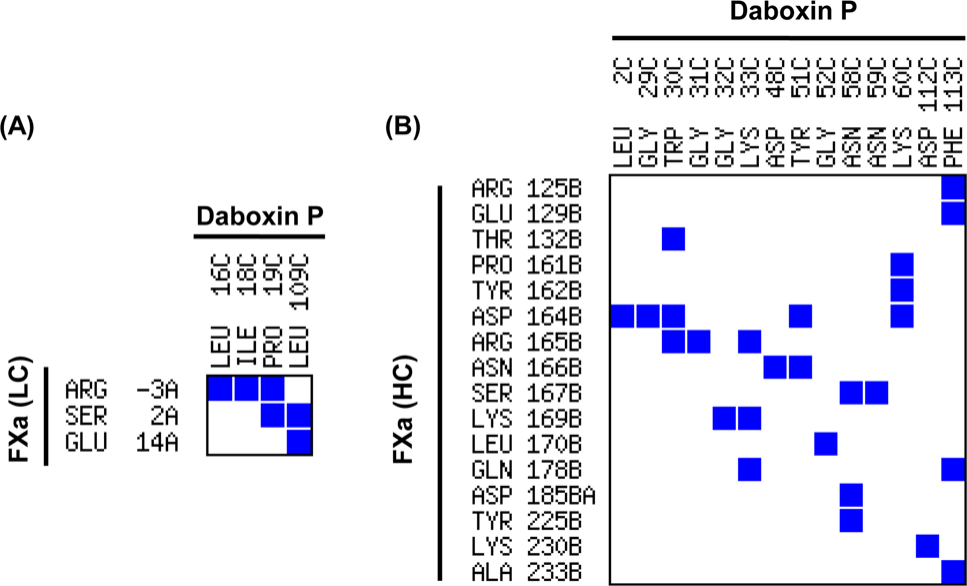
Contact map of daboxin P-FXa complex generated using online server Contact Map Analysis. **(A):** residue to residue contact of light chain of FXa and daboxin P **(B):** residue to residue contact of heavy chain of FXa and daboxin P. The residue to residue contact area of the interacting amino acid residues for the chains has been considered above 8 Å^2^ for the design of the contact map.

Contact map analysis shows fourteen residues of daboxin P share a contact area greater than 8 Å^2^ with sixteen residues of the heavy chain of FXa while only four residues of daboxin P are found to share a contact area greater than the threshold limit (8 Å^2^) with three residues of the light chain of FXa. This suggests the interaction of daboxin P with the light and heavy chain of FXa (Fig 13) (Table C in S1 File).

## Discussion

Snake venom is a plethora of pharmacologically active components with plausible therapeutic relevance in the treatment of coagulopathic abnormalities, hypotension and as diagnostic tools. Anticoagulant proteins are one such components of the venom mostly sought for the treatment of thrombosis and thromboembolism. Daboxin P, a non-toxic PLA_2_ enzyme purified from the venom of Indian *Daboia r*. *russelii* exhibits strong anticoagulant activity under *in-vitro* and *in-vivo* conditions. Dissection of the coagulation cascade reveals its effect upstream of the common pathway. However, it did not exhibit inhibitory effect on the amidolytic activity of the tested serine proteases. Nonetheless, daboxin P inhibited the activation of FX to FXa by both the tenase complexes in the presence and absence of phospholipids suggesting its enzymatic and non-enzymatic mode of action. Apart from phospholipids and calcium ions, FX is common in the tenase complexes hence daboxin P might be interacting with FX to exhibit its non-enzymatic mechanism of anticoagulant activity. Fluorescence emission spectroscopy supports this hypothesis where we observed the quenching of emission spectra of FX upon incubation with daboxin P for different time intervals. Apart from inhibiting the tenase complexes, daboxin P also inhibited the thrombin formation by the prothrombinase complex when pre-incubated with FXa in presence of phospholipids and Ca^+2^ ions followed by addition of FVa. Thus, daboxin P might interact with FXa to a region other than the active site or inhibit the complex formation by creating a steric hindrance. The quenched fluorescence emission spectra of FXa upon incubation with daboxin P suggest the probable interaction of daboxin P with FXa for exhibiting its anticoagulant activity non-enzymatically. This interaction is further supported by the affinity chromatography studies where FX and FXa were coupled to daboxin P immobilized CNBr resin and eluted at 1 M NaCl.

CM-IV, a PLA_2_ enzyme isolated from the venom of *Naja nigricollis* exhibits anticoagulant activity by inhibiting both the extrinsic tenase complex and prothrombinase complex [6,15]. It interacts with FVIIa and FXa through its anticoagulant region which shares partial sequence similarity to a region of tissue factor and partly to the light chain of FVa respectively [6,15]. However, the anticoagulant region of daboxin P does not share any such sequence similarity with FVa or TF, suggesting a different mechanism of action for daboxin P to exhibit its anticoagulant effect. Ammodytoxin A (AtxA), a PLA_2_ enzyme from the venom of *Vipera ammodytes ammodytes* inhibits prothrombinase complex by binding to FVa binding site on FXa [22]. Sequence alignment of daboxin P with AtxA shows ~76% sequence similarity with minor substitutions at helix A, helix B, Ca^+2^ binding loop, β-wing, helix D and C-terminal region (Figure D in S1 File). Such variations in the amino acid sequence of the PLA_2_ enzymes are well documented in literature which are known to confer wide range of pharmacological specificity to these proteins towards various physiological targets [65].

Based on molecular docking analysis, Faure and co-workers reported the interaction of the heavy chain of FXa with the Ca^+2^ binding loop, helix C, β-wing and the C-terminal region of AtxA, while light chain of FXa is involved in interaction with helix A, B and the β-wing [3,22]. On the other hand, the present *in-silico* molecular docking analysis shows the interaction of the Ca^+2^ binding loop (Trp30, Gly31, Gly32); helix C (Asp48, Tyr 51 and Gly52), anticoagulant region (Asn58) and C-terminal region (Phe113) of daboxin P with the heavy chain of FXa (Thr132, Arg165, Lys169, Asn166, Leu170, Tyr225 and Arg125 of FXa) but not with its light chain. The residues of helix A (Leu3, Met7, Leu10), helix B (Asn16, Pro17, Leu18, Thr19) and the anticoagulant region (Arg68) of AtxA are reported to be involved in the interaction with the light chain of FXa [22]. However, most of these crucial residues were found to be substituted in daboxin P (Lys7, Leu16, Ala17, Ile18, Pro19, Lys68) due to which daboxin P might not have shown any interaction with the light chain of FXa in the docking study. Nevertheless, contact map analysis shows interaction of the light chain of FXa with helix B (Leu16, Ile18, Pro19) and C-terminal region (Leu109) of daboxin P while the heavy chain of FXa (Arg125B, Glu129B, Thr132B, Pro161B, Tyr162B, Asp164B, Arg165B, Asn166B, Ser167B, Lys169B, Leu170B, Gln178B, Asp185B, Tyr225B, Lys230B, Ala233B) is found to interact with helix A (Leu2), Ca^+2^ binding loop (Gly29, Trp30, Gly31, Gly32, Lys33), helix C (Asp48, Tyr51, Gly52), anticoagulant region (Asn58, Asn59, Lys60), and the C-terminal region (Asp112, Phe113) of daboxin P (Fig 13).

It has been reported that Arg165, Lys169 and Lys230 of FXa form the core for FVa binding site while Arg125 and Glu129 are crucial for the complex formation [64]. Hence, binding of daboxin P to FXa might hinder the association of FXa and FVa for the formation of the prothrombinase complex in presence or absence of other co-factors. Norledge B.V. and colleagues reported the involvement of Asp4 and Asp7 of EGF-II of light chain and Asp185a, Lys186 and Lys134 of heavy chain of FXa for binding to TF/FVIIa complex based on site directed mutagenesis and molecular docking [66]. With docking analysis it has been observed that daboxin P binds to FXa (discussed above) in close vicinity to FVIIa-TF binding region on FX. Thus, interaction of daboxin P with those proximal regions might create a steric hindrance in light chain and heavy chain regions of FX and inhibit the binding of FVIIa-TF or FIXa-FVIIIa. The residues involved in this interaction could not be confirmed by docking analysis as the crystal structure of FX is not available. Based on the biochemical, biophysical and docking analysis, it is proposed that daboxin P might be exhibiting its anticoagulant activity by interacting with FX and FXa. However, site-directed mutagenesis of the critical amino acids residues needs to be carried out to confirm the interaction of daboxin P with FX and FXa.

Thus, present study evaluates daboxin P as a probable natural inhibitor of FX and FXa from snake venom. FXa is one of the most pivotal components of the physiological system which plays critical role not only in haemostasis but also in intracellular signal transduction leading to pathophysiological events like fibrosis, cancer and tissue modelling restoration [67]. Hence, thriving for natural FX/FXa inhibitors will eventually lead to manipulation/management of various pathophysiological aspects of human system.

## Supporting Information

S1 File **Table A. Peptide fragments of daboxin P obtained from tandem mass spectrometry**. **MH+** stands for mass/charge (m/z) of the peptide (protonated molecular ions), **Z** represents the number of charges a peptide carries after ionization, **Score** implies the sum of all peptide cross correlation (Xcorr) values. **Figure A. The Phylogenetic relationship of daboxin P with the reported anticoagulant PLA**_**2**_ **enzymes from snake venom.** The phylogenetic tree was constructed by neighbour joining (NJ) tree using Mega 5 software with a bootstrap value of 1000. Anticoagulant PLA_2_ enzymes with complete sequence were obtained from Pubmed Protein Database (http://www.ncbi.nlm.nih.gov/pubmed) and aligned using ClustalW. The sequences used in the study were CBc (*Crotalus durissus terrificus*), CB crotoxin (*Crotalus durissus collilineatus*), CBb (*Crotalus durissus terrificus*), PLA2 F17 (*Crotalus durissus terrificus*), CBa2 (*Crotalus durissus terrificus*), Cdc-9 (*Crotalus durissus cumanensis*), Cdc-10 (*Crotalus durissus cumanensis*), bID-PLA2 (*Bothrops leucurus*), Bothropstoxin (*Bothrops jararacussu*), DPLA2 (*Daboia russellii russellii*), vurtoxin (*Vipera renardi*), AtxA (*Vipera ammodytes ammodytes*), AtxC (*Vipera ammodytes ammodytes*), bAhp (*Gloydius halys*), BP III (*Protobothrops flavoviridis*), II-BP (*Protobothrops flavoviridis*), Ts-K49b (*Trimeresurus stejneger*i), MTxII (*Bothrops asper*), PLA2 homology (*Bothrops atrox*), BthA-I-PLA2 (*Bothrops jararacussu*), Vur PL2B (*Vipera renardi*), EC-I-PLA2 (*Echis carinatus*), PLa2Vb (*Vipera berus berus*), CbII (*Pseudocerastes fieldi*), HDP-1P (*Vipera nikolskii*), HD-2P (*Vipera nikolskii*), Caudoxin (*Bitis caudalis*), APLA2-2 (*Ophiophagus hannah*); CM-III/CM-IV (*Naja nigricollis*); CM-III (*Naja mossambica*); CM-II (*Naja mossambica*) and CM-I (*Naja mossambica*).**Figure B. Effect of daboxin P on thrombin time and fibrinogen. (i):** Different amount of daboxin P (1, 3, 5 μg) were pre-incubated with 50 μl platelet poor plasma for 3 min at 37°C. 50 μl of thrombin (10 u/ml) was added to initiate clot formation and monitored on Tulip Coastat-1 coagulo analyser. Clotting time in presence of Tris buffer (20 mM, pH 7.4) was taken as normal clotting time (NCT). The results are mean ± SD of three independent experiments. **(ii):** Assessment of fibrinogenolytic activity on SDS-PAGE, different amount of daboxin P (0.01, 0.1, 1, 5 μg) were pre-incubated with 300 μl of 2 mg/ml fibrinogen for 24 h at 37°C. Fibrinogen with thrombin (3 μl of 10 units/ml) was considered as the positive control and fibrinogen with buffer (50 mM Tris-Cl, pH 7.5) was taken as the negative control. Fibrinogen degradation products were analyzed on 12.5% glycine SDS-PAGE, stained with CBB R-250. (C: Fibrinogen, Thr: Thrombin, Rvv: crude Russell’s viper venom). **Figure C. Three dimensional (3D) molecular modelling (i):** The 3D ribbon structure of daboxin P (in red, blue, green & white) was superimposed with X-ray crystallographic structure of AtxA (PDB 3G8G) (in yellow), **(ii):** 3D ribbon model of daboxin P as predicted by I-TASSER server. The Ca^+2^ binding loop (YGCYCGCGG in torquise blue), anticoagulant region (53NLPDCNNKSKRYRYKK68) (in yellow) and the active site His residue (in stick) are highlighted. **Table B. Summary of interface plot statistics of the docked model of daboxin P and FXa. Table C. List of displayed residue to residue contact area of the light (A) and heavy chain (B) of FXa with daboxin P (C). Figure D. Sequence alignment of daboxin P with ammodytoxin A.** The amino acid residues varying in daboxin P with respect to ammodytoxin A are underlined. The predicted anticoagulant region in both the PLA_2_ enzymes is highlighted in red.
(DOCX)
