# Supplementary material for: Daboxin P, a Major Phospholipase A2 Enzyme from the Indian Daboia russelii russelii Venom Targets Factor X and Factor Xa for Its Anticoagulant Activity
Source: PLoS One. 2016 Apr 18;11(4):e0153770. doi: 10.1371/journal.pone.0153770 (PMC4835082; doi:10.1371/journal.pone.0153770)
Supplement: S1 File — Table A. Peptide fragments of daboxin P obtained from tandem mass spectrometry. MH+ stands for mass/charge (m/z) of the peptide (protonated molecular ions), Z represents the number of charges a peptide carries after ionization, Score implies the sum of all peptide cross correlation (Xcorr) values. Figure A. The Phylogenetic relationship of daboxin P with the reported anticoagulant PLA2 enzymes from snake venom. The phylogenetic tree was constructed by neighbour joining (NJ) tree using Mega 5 software with a bootstrap value of 1000. Anticoagulant PLA2 enzymes with complete sequence were obtained from Pubmed Protein Database (http://www.ncbi.nlm.nih.gov/pubmed) and aligned using ClustalW. The sequences used in the study were CBc (Crotalus durissus terrificus), CB crotoxin (Crotalus durissus collilineatus), CBb (Crotalus durissus terrificus), PLA2 F17 (Crotalus durissus terrificus), CBa2 (Crotalus durissus terrificus), Cdc-9 (Crotalus durissus cumanensis), Cdc-10 (Crotalus durissus cumanensis), bID-PLA2 (Bothrops leucurus), Bothropstoxin (Bothrops jararacussu), DPLA2 (Daboia russellii russellii), vurtoxin (Vipera renardi), AtxA (Vipera ammodytes ammodytes), AtxC (Vipera ammodytes ammodytes), bAhp (Gloydius halys), BP III (Protobothrops flavoviridis), II-BP (Protobothrops flavoviridis), Ts-K49b (Trimeresurus stejnegeri), MTxII (Bothrops asper), PLA2 homology (Bothrops atrox), BthA-I-PLA2 (Bothrops jararacussu), Vur PL2B (Vipera renardi), EC-I-PLA2 (Echis carinatus), PLa2Vb (Vipera berus berus), CbII (Pseudocerastes fieldi), HDP-1P (Vipera nikolskii), HD-2P (Vipera nikolskii), Caudoxin (Bitis caudalis), APLA2-2 (Ophiophagus hannah); CM-III/CM-IV (Naja nigricollis); CM-III (Naja mossambica); CM-II (Naja mossambica) and CM-I (Naja mossambica).Figure B. Effect of daboxin P on thrombin time and fibrinogen. (i): Different amount of daboxin P (1, 3, 5 μg) were pre-incubated with 50 μl platelet poor plasma for 3 min at 37°C. 50 μl of thrombin (10 u/ml) was added to initiate clo [file pone.0153770.s001.docx]

**Supplementary figures and tables**

**Table A. Peptide fragments of daboxin P obtained from tandem mass spectrometry**. **MH+** stands for mass/charge (m/z) of the peptide (protonated molecular ions), **Z** represents the number of charges a peptide carries after ionization, **Score** implies the sum of all peptide cross correlation (Xcorr) values.

| **Sl.No.** | **MS/MS sequence** | **No.of peptides** | **MH+ (Da)** | **Z** | **Coverage & score** |
| --- | --- | --- | --- | --- | --- |
| 1. | YMLYPDFLcKGELK | 7 | 1776.14 | 3 | 80.17  &  31.65 |
| 2. | VNGAIVcEK | 8 | 989.36 | 1 |  |
| 3. | SLLEFGKMILEETGK | 7 | 1695.85 | 3 |  |
| 4. | GTSCENTRICECDK | 7 | 1457.00 | 2 |  |
| 5. | QNLNTYSK | 7 | 967.47 | 1 |  |
| 6. | MILEETGK | 10 | 920.47 | 1 |  |
| 7. | LAIPSYSSYGCYCGWGGK | 7 | 1911.51 | 3 |  |
| 8. | KYmLYPDFLcK | 7 | 1494.36 | 2 |  |
| 9. | IcEcDK | 11 | 824.27 | 1 |  |
| 10. | AAAIcFR | 23 | 808.40 | 1 |  |
| 11. | IcEcDKAAAIcFRQNLNTYSK | 8 | 2504.97 | 3 |  |

**Figure A: The Phylogenetic relationship of daboxin P with the reported anticoagulant PLA_2_ enzymes from snake venom.** The phylogenetic tree was constructed by neighbour joining (NJ) tree using Mega 5 software with a bootstrap value of 1000. Anticoagulant PLA_2_ enzymes with complete sequence were obtained from Pubmed Protein Database (http://www.ncbi.nlm.nih.gov/pubmed) and aligned using ClustalW. The sequences used in the study were CBc (*Crotalus durissus terrificus*), CB crotoxin (*Crotalus durissus collilineatus*), CBb (*Crotalus durissus terrificus*), PLA2 F17 (*Crotalus durissus terrificus*), CBa2 (*Crotalus durissus terrificus*), Cdc-9 (*Crotalus durissus cumanensis*), Cdc-10 (*Crotalus durissus cumanensis*), bID-PLA2 (*Bothrops leucurus*), Bothropstoxin (*Bothrops jararacussu*), DPLA2 (*Daboia russellii russellii*), vurtoxin (*Vipera renardi*), ATxA (*Vipera ammodytes ammodytes*), ATxC (*Vipera ammodytes ammodytes*), bAhp (*Gloydius halys*), BP III (*Protobothrops flavoviridis*), II-BP (*Protobothrops flavoviridis*), Ts-K49b (*Trimeresurus stejneger*i), MTxII (*Bothrops asper*), PLA2 homology (*Bothrops atrox*), BthA-I-PLA2 (*Bothrops jararacussu*), Vur PL2B (*Vipera renardi*), EC-I-PLA2 (*Echis carinatus*), PLa2Vb (*Vipera berus berus*), CbII (*Pseudocerastes fieldi*), HDP-1P (*Vipera nikolskii*), HD-2P (*Vipera nikolskii*), Caudoxin (*Bitis caudalis*), APLA2-2 (*Ophiophagus hannah*); CM-III/CM-IV (*Naja nigricollis*); CM-III (*Naja mossambica*); CM-II (*Naja mossambica*) and CM-I (*Naja mossambica*).

**Figure B. Effect of daboxin P on thrombin time and fibrinogen. (i):** Different amount of daboxin P (1, 3, 5 µg) were pre-incubated with 50 µl platelet poor plasma for 3 min at 37^o^C. 50 µl of thrombin (10 u/ml) was added to initiate clot formation and monitored on Tulip Coastat-1 coagulo analyser. Clotting time in presence of Tris buffer (20 mM, pH 7.4) was taken as normal clotting time (NCT). The results are mean ± SD of three independent experiments. **(ii):** Assessment of fibrinogenolytic activity on SDS-PAGE, different amount of daboxin P (0.01, 0.1, 1, 5 µg) were pre-incubated with 300 µl of 2 mg/ml fibrinogen for 24 h at 37°C. Fibrinogen with thrombin (3 µl of 10 units/ml) was considered as the positive control and fibrinogen with buffer (50 mM Tris-Cl, pH 7.5) was taken as the negative control. Fibrinogen degradation products were analyzed on 12.5% glycine SDS-PAGE, stained with CBB R-250. (C: Fibrinogen, Thr: Thrombin, Rvv: crude Russell’s viper venom).

**Figure C. Three dimensional (3D) molecular modelling (i):** The 3D ribbon structure of daboxin P (in red, blue, green & white) was superimposed with X-ray crystallographic structure of AtxA (PDB 3G8G) (in yellow), **(ii):** 3D ribbon model of daboxin P as predicted by I-TASSER server. The Ca^+2^ binding loop (YGCYCGCGG in torquise blue), anticoagulant region (53NLPDCNNKSKRYRYKK68) (in yellow) and the active site His residue (in stick) are highlighted.

**Table B. Summary of interface plot statistics of the docked model of daboxin P and FXa.**

**Table C. List of displayed residue to residue contact area of the light (A) and heavy chain (B) of FXa with daboxin P (C).**

| Interacting chains | Residue 1 | Residue 2 | Contact Area (Å^2^) |
| --- | --- | --- | --- |
| A-C | ARG -3A | LEU -16C | 24.300 |
|  | ARG -3A | ILE -18C | 24.700 |
|  | ARG -3A | PRO -19C | 54.000 |
|  | SER -2A | PRO -19C | 9.000 |
|  | SER -2A | LEU -109C | 13.500 |
|  | GLU -14A | LEU -109C | 17.900 |
| B-C | ARG -125B | PHE -113C | 42.000 |
|  | GLU -129B | PHE -113C | 11.900 |
|  | THR -132B | TRP -30C | 56.100 |
|  | PRO -161B | LYS -60C | 10.500 |
|  | TYR -162B | LYS -60C | 11.300 |
|  | ASP -164B | LEU -2C | 10.700 |
|  | ASP -164B | GLY -29C | 13.900 |
|  | ASP -164B | TRP -30C | 15.600 |
|  | ASP -164B | TYR -51C | 9.400 |
|  | ASP -164B | LYS -60C | 24.200 |
|  | ARG -165B | TRP -30C | 30.300 |
|  | ARG -165B | GLY -31C | 15.900 |
|  | ARG -165B | LYS -33C | 10.100 |
|  | ASN -166B | ASP -48C | 54.500 |
|  | ASN -166B | TYR -51C | 20.400 |
|  | SER -167B | ASN -58C | 17.900 |
|  | SER -167B | ASN -59C | 18.800 |
|  | LYS -169B | GLY -32C | 27.800 |
|  | LYS -169B | LYS -33C | 10.300 |
|  | LEU -170B | GLY -52C | 48.200 |
|  | GLN -178B | LYS -33C | 24.600 |
|  | GLN -178B | PHE -113C | 16.900 |
|  | ASP -185BA | ASN -58C | 25.500 |
|  | TYR -225B | ASN -58C | 34.500 |
|  | LYS -230B | ASP -112C | 10.300 |
|  | ALA -233B | PHE -113C | 18.900 |

**Figure D. Sequence alignment of daboxin P with ammodytoxin A.** The amino acid residues varying in daboxin P with respect to ammodytoxin A are underlined. The predicted anticoagulant region in both the PLA_2_ enzymes are highlighted in red.
